# Cognitive and academic benefits of music training with children: A multilevel meta-analysis

**DOI:** 10.3758/s13421-020-01060-2

**Published:** 2020-07-29

**Authors:** Giovanni Sala, Fernand Gobet

**Affiliations:** 1 Institute for Comprehensive Medical Science (ICMS), Fujita Health University, Toyoake, Aichi Japan; 2 Centre for Philosophy of Natural and Social Science, London School of Economics and Political Science, London, WC2A 2AE UK

**Keywords:** Academic achievement, Cognitive ability, Cognitive training, Music, Transfer

## Abstract

Music training has repeatedly been claimed to positively impact children’s cognitive skills and academic achievement (literacy and mathematics). This claim relies on the assumption that engaging in intellectually demanding activities fosters particular domain-general cognitive skills, or even general intelligence. The present meta-analytic review (*N* = 6,984, *k* = 254, *m* = 54) shows that this belief is incorrect. Once the quality of study design is controlled for, the overall effect of music training programs is null ($\overline{g}$ ≈ 0) and highly consistent across studies (*τ*^*2*^ ≈ 0). Results of Bayesian analyses employing distributional assumptions (informative priors) derived from previous research in cognitive training corroborate these conclusions. Small statistically significant overall effects are obtained only in those studies implementing no random allocation of participants and employing non-active controls ($\overline{g}$ ≈ 0.200, *p* < .001). Interestingly, music training is ineffective regardless of the type of outcome measure (e.g., verbal, non-verbal, speed-related, etc.), participants’ age, and duration of training. Furthermore, we note that, beyond meta-analysis of experimental studies, a considerable amount of cross-sectional evidence indicates that engagement in music has no impact on people’s non-music cognitive skills or academic achievement. We conclude that researchers’ optimism about the benefits of music training is empirically unjustified and stems from misinterpretation of the empirical data and, possibly, confirmation bias.

**Electronic supplementary material:**

The online version of this article (10.3758/s13421-020-01060-2) contains supplementary material, which is available to authorized users.

## Introduction

It has been claimed that music fosters children’s cognitive skills and academic achievement. Learning to play the violin or the piano, to recognize pitches, and to keep the beat are often presented as effective cognitive enhancement tools (e.g., Jaušovec & Pahor, 2017). However, the idea that practicing cognitively demanding tasks may lead to domain-general cognitive enhancement is in stark contrast with empirical evidence in cognitive science and educational psychology. In fact, while human cognition has been shown to be malleable to training, transfer of skills appears to be limited to the training domain and, at best, other similar domains.

It is customary to distinguish between two broad categories of transfer: near transfer and far transfer (Barnett & Ceci, 2002). Whereas near transfer – i.e., the transfer of skills within the same domain – is sometimes observed, far transfer – i.e., the transfer of skills across two distant domains – is rare or possibly inexistent (Melby-Lervåg, Redick, & Hulme, 2016, Sala et al., 2019a). Moreover, when it does occur, transfer of skills is often limited to the degree to which the two domains (source and target) share contents. For example, even transfer of skills within subspecialties of the same discipline seems to be limited. In fact, performance significantly worsens when experts engage in certain subspecialties of their field of expertise. For example, chess masters who are asked to recall or find the best move in positions coming from chess openings that do not fall into their repertoire exhibit a drastic (about 1 SD) reduction in performance (Bilalić, McLeod, & Gobet, 2009). This so-called *curse of specificity* has been recently defined as one of the fundamental particles in the standard model of human cognition (Sala & Gobet, 2019).

Researchers involved in cognitive training do not deny that between-domain, or even within-domain, transfer is hard to trigger. Nonetheless, they claim that it is possible to induce far transfer by engaging in domain-specific cognitively demanding activities that boost domain-general cognitive skills; those skills, in turn, are supposed to generalize across many different domains (e.g., academic proficiency; Strobach & Karbach, 2016). At a neural level, this generalization is thought to be enabled by the activation of shared brain structures that are common to the practiced activity (e.g., music) and other core cognitive skills (e.g., fluid intelligence, working memory, and language; Moreno et al., 2011). In other words, domain-general cognitive enhancement and far transfer are believed to be by-products of domain-specific training (Taatgen, 2016).

With respect to music, three main hypotheses have been formulated to explain why playing it should lead to broad cognitive benefits. To begin with, music might directly impact on general intelligence rather than on some particular cognitive skills (Schellenberg, 2004). This idea is consistent with the vast amount of correlational evidence showing that musicians tend to outperform non-musicians in a variety of cognitive tests. Examples include memory (Sala & Gobet, 2017a; Talamini, Altoè, Carretti, & Grassi, 2017), fluid and general intelligence (Ruthsatz, Detterman, Griscom, & Cirullo, 2008; Schellenberg, 2006), attention (Saarikivi, Putkinen, Tervaniemi, & Huotilainen, 2016), and phonological processing (Forgeard et al., 2008). The same pattern of results occurs in academic skills. In fact, music skills appear to be related to better reading abilities (Anvari, Trainor, Woodside, & Levy, 2002), and music engagement is a predictor of overall academic achievement (Wetter, Koerner, & Schwaninger, 2009).

Another possible link connecting music engagement and cognitive enhancement might be working memory (WM). Multimodal cognitively demanding activities are thought to strengthen WM capacity (Diamond & Ling, 2019; Morrison & Chein, 2011), which, in turn, enhances fluid intelligence and learning (Jaeggi et al., 2008). Music training is one such activity (Saarikivi, Huotilainen, Tervaniemi, & Putkinen, 2019). Simply put, the putative broad benefits of music training would stem from a boost in domain-general WM capacity rather than general intelligence.

Finally, music training might positively impact on one’s sound perception and, consequently, phonological processing and even reading skills (Patel, 2011; Tierney & Kraus, 2013). This hypothesis is upheld by the fact that numerous brain structures and neural patterns are shared by music skills and language (for a review, see Jäncke, 2009). Interestingly, improved reading skills may also facilitate the acquisition of new skills and therefore enhance people’s IQ performance (Ritchie & Tucker-Drob, 2018). This further mechanism would again be consistent with the overall idea that music training conveys multiple cognitive and academic benefits.

### Experimental evidence

The theories just described imply that music training *causes* cognitive enhancement and improvement in academic performance. However, correlational evidence gathered in natural groups is not sufficient to establish a causal link. In the last few decades, dozens of experimental trials have been carried out to examine a potential causal link between music training and improved cognitive/academic performance.

Researchers in this field have reached inconsistent conclusions. While most of them have expressed optimism about the benefits of music training (e.g., Barbaroux, Dittinger, & Besson, 2019; Nan et al., 2018; Tierney, Krizman, & Kraus, 2015), others have found this enthusiasm unjustified (e.g., Kempert et al., 2016; Rickard, Bambrick, & Gill, 2012). Like in many other fields in the social sciences, meta-analyses have been carried out to resolve such controversies. The only comprehensive meta-analytic review performed so far about the benefits of music training is that by Sala and Gobet (2017b). This meta-analysis – which includes 38 studies, 118 effect sizes, and 3,085 participants – found an overall effect of $\overline{d}$= 0.16. It also highlighted that the impact of music training on cognitive skills and academic performance was a function of the quality of the study’s experimental design. Specifically, the magnitude of the music-induced effects was significantly smaller (around zero) in those studies implementing active controls and random allocation of the participants to groups.

Two meta-analyses examined a subset of studies (Cooper, 2019; Gordon, Fehd, & McCandliss, 2015), and drew somewhat more positive implications for the cognitive and educational benefits of music teaching. Gordon et al. (2015) reviewed 12 studies (*n* = 901) assessing the effects of music training on language-related skills. The overall effect was small but significant ($\overline{d}$ = 0.20). Analogously, Cooper (2019) analysed 21 studies (*n* = 1,767) and found an overall effect size of $\overline{g}$ = 0.28 across several measures of cognitive ability (measures related to academic achievement were not included because they were considered too different from cognitive ability). Interestingly, the effect was maintained in studies employing active controls ($\overline{g}$ = 0.21).

### The present meta-analysis

Despite the less than encouraging evidence, dozens of new experimental investigations have been carried out in recent years, including the two largest randomized control trials (RCTs) in this field (Aleman et al., 2017; Haywood et al., 2015). Once again, the claims about the effectiveness of music training have been inconsistent across studies (e.g., James et al., 2019; Lukács & Honbolygó, 2019; Nan et al., 2018). We thus ran a meta-analysis including both old and new experimental studies to establish (a) which claims are justified, (b) what are the sources of heterogeneity across studies, and (c) which of the theories predicting that music training enhances cognitive and academic skills are corroborated/refuted.

Beyond being relatively dated, the previous meta-analyses suffer from several technical limitations. First, no multilevel modeling was employed. Multilevel modeling is necessary to adjust standard errors when a certain degree of statistical dependence is present in the data (i.e., effect sizes nested in studies).^1^ Also, some of the effect sizes were incorrectly calculated because of a mistake in the reporting of the results in one of the primary studies (Rickard et al., 2012; personal communication). Both issues probably inflated the amount of between-study true heterogeneity, which tended to bias meta-analytic model estimates. In addition, the presence of a non-negligible amount of unexplained true heterogeneity (as in both Sala & Gobet, 2017b, and Cooper, 2019) makes the overall effect sizes hard to interpret because the sources of between-study variability remain hidden. Finally, no thorough sensitivity analysis was performed (e.g., outlier analysis and multiple publication bias analysis). In brief, such suboptimal modeling choices produce biased estimates. The present meta-analytic review aims to correct these problems and to update the findings of the music-training literature. The current meta-analysis also carries out Bayesian analyses that compare the support for the null and alternative hypotheses, and relies on a larger number of studies (19 new studies) and therefore a larger number of participants (an increase from about 3,000 to about 7,000, compared to Sala & Gobet, 2017b). Since the number of participants and effect sizes are more than double, the current meta-analysis has a much higher power than the 2017 meta-analysis.

## Method

### Literature search

A systematic search strategy was implemented (Appelbaum et al., 2018). Using the following Boolean string (“music” OR “musical”) AND (“training” OR “instruction” OR “education” OR “intervention”), we searched through ERIC, PsycINFO, and ProQuest Dissertation & Theses databases to find studies that reported music training programs. We retrieved 3,044 records.

### Inclusion criteria

Five inclusion criteria were applied:

1. The study was experimental in nature and implemented a cognitively demanding music-training program (e.g., learning to play instruments, Kodály method, etc.). No correlational or ex-post facto studies were included.
2. The study included at least one control group that isolated the variable of interest (i.e., music training).
3. The study included non-music-related cognitive tests or academic outcomes.
4. The study included participants aged between 3 and 16 years with no previous formal music experience or clinical condition.
5. The study reported sufficient data to calculate the effect sizes. Alternatively, the author(s) had to provide the necessary data.

**Fig. 1:**
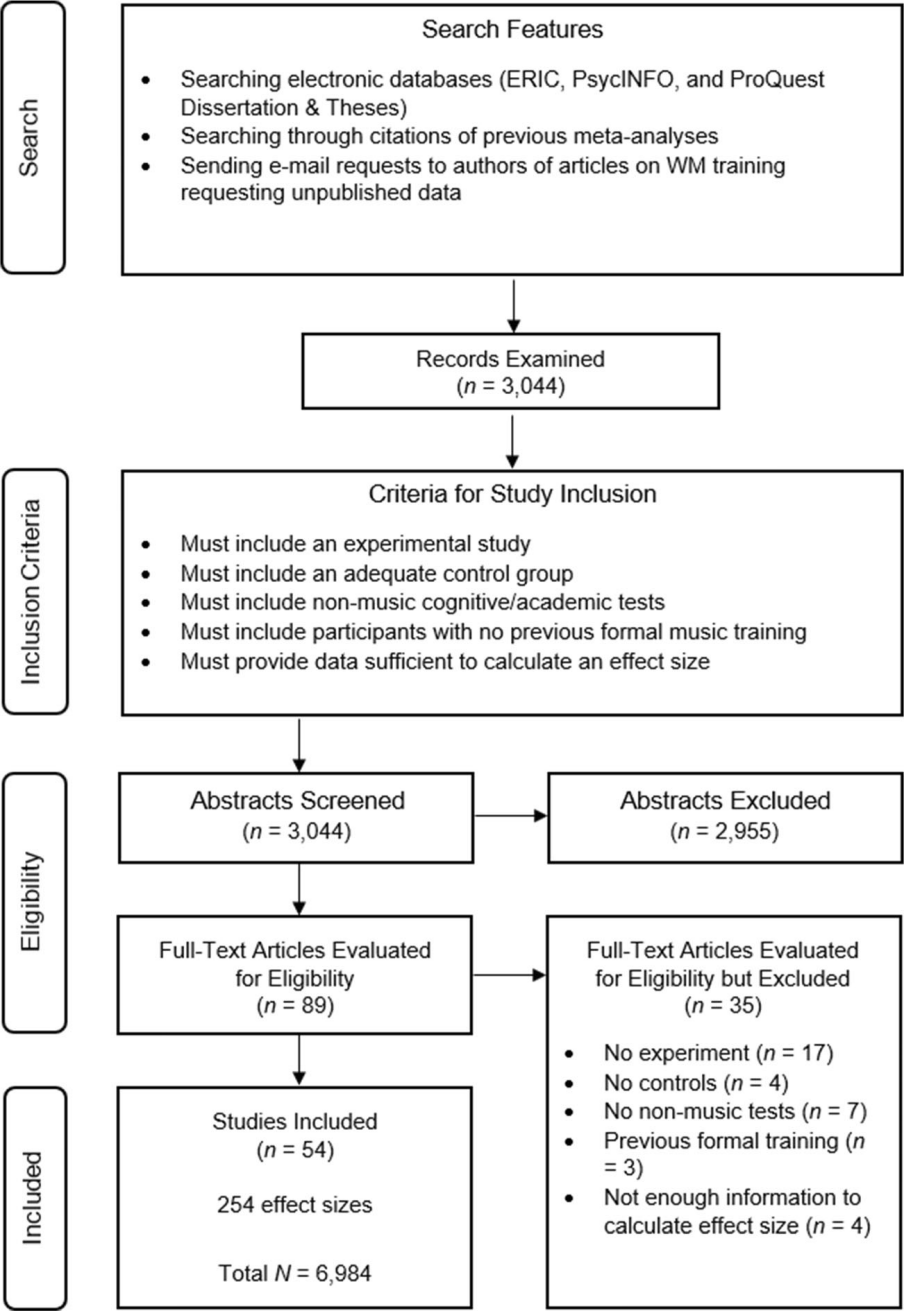
Flow diagram of the search strategy

We searched for eligible articles through 1 December 2019. When the data reported in the study were insufficient to calculate the effect sizes or important details about the study design were unclear, we contacted the corresponding authors by email (*n* = 11). We received three positive replies. We found 54 studies, conducted from 1986 to 2019, that met the inclusion criteria (reported in Appendix A in the Supplemental Online Materials). Nineteen of these studies had never been included in any previous meta-analysis. The studies included 254 effect sizes and a total of 6,984 participants. Thus, compared to the previous most comprehensive meta-analysis in the field (i.e., Sala & Gobet, 2017b), the number of both effect sizes and participants was more than doubled. The studies originally evaluated for inclusion but eventually excluded are reported in Appendix B in the Supplemental Online Materials. The procedure is described in Fig. 1.

### Moderators

We assessed six moderators based on the previous meta-analyses in the literature:

1. Baseline difference (continuous variable): The standardized mean difference between the experimental and control groups at pre-test. This moderator was added to evaluate the amount of true heterogeneity accounted for by pre-post-test regression to the mean. It thus aimed at ruling out potential confounding effects of this statistical artifact.
2. Randomization (dichotomous variable): Whether the children were randomly allocated to the groups.
3. Type of controls (active or non-active; dichotomous variable): Whether the music training group was compared to another novel activity (e.g., dancing); no-contact groups and business-as-usual groups were treated as “non-active.” This moderator thus controlled for potential placebo effects.
4. Age (continuous variable): The mean age of the study’s participants. A few studies did not report the participants’ mean age. In these cases, the participants’ mean age was obtained from the median or the school grade.
5. Outcome measure: The effect sizes were grouped into four broad groups based on the Cattell-Horn-Carroll taxonomy (McGrew, 2009): *non-verbal ability* (e.g., fluid reasoning [Gf], mathematical skills [Gq], and spatial skills [Gv]); *verbal ability* (e.g., vocabulary and reading skills [Gc], phonological processing [Grw]); *memory* (e.g., short-term/working-memory tasks [Gsm]); and *speed* (e.g., processing speed [Gs] and inhibition tasks [Gt]). The inter-rater agreement was *κ* = 1. We also examined this moderator without grouping these eight categories into the four groups. Finally, since some primary studies employed academic tests (e.g., Haywood et al., 2015), we examined whether the effect sizes related to cognitive skills were greater than those related to academic achievement (as suggested by Cooper, 2019). The latter category included all those effect sizes that were obtained from academic tests of literacy and mathematics (a subset of the Gc and Gq groups). All the other effect sizes fell into the cognitive group.
6. Duration of training: The total number of hours, weeks, and sessions of the training program in each study. These three variables were tested separately because they were collinear (i.e., they measured the same construct with three different metrics).

### Effect size calculation

The effect sizes were calculated for each eligible outcome measure in the primary studies. Hedges’s *g*s – an adjusted standardized mean difference – were calculated with the following formula:

1 $$ g=d\times \left(1-\frac{3}{\left(4\times N\right)-9}\right) $$

with

2 $$ d=\frac{\left({M}_{e\_ post}-{M}_{e\_ pre}\right)-\left({M}_{c\_ post}-{M}_{c\_ pre}\right)}{S{D_{pooled}}_{pre}} $$

where *M*_*e_post*_ and *M*_*e_pre*_ are the mean of the experimental group at post-test and pre-test, respectively, *M*_*c_post*_ and *M*_*c_pre*_ are the mean of the control group at post-test and pre-test, respectively, *SD*_*pooled_pre*_ is the pooled pre-test SDs in the experimental group and the control group, and *N* is the total sample size.

The sampling error variances were calculated with the following formula:

3 $$ Va{r}_g=\left(\frac{N_e-1}{N_e-3}\times \left(\frac{2\times \left(1-r\right)}{r_{xx}}+\frac{d_e^2}{2}\times \frac{N_e}{N_e-1}\right)\times \frac{1}{N_e}+\frac{N_c-1}{N_c-3}\times \left(\frac{2\times \left(1-r\right)}{r_{xx}}+\frac{d_c^2}{2}\times \frac{N_c}{N_c-1}\right)\times \frac{1}{N_c}\right)\times {\left(1-\frac{3}{\left(4\times N\right)-9}\right)}^2 $$

where *r*_*xx*_ is the test-retest reliability of the test, *N*_*e*_ and *N*_*c*_ are the sample sizes of the experimental group and the control group, respectively, *d*_*e*_ and *d*_*c*_ are the within-group standardized mean differences of the experimental group and the control group, respectively. Finally, *r* is the pre-post-test correlation (Schmidt & Hunter, 2015; pp. 343–355). The pre-post-test correlations and test-retest coefficients were rarely provided in the primary studies. Therefore, we assumed the reliability coefficient (*r*_*xx*_) to be equal to the pre-post-test correlation (i.e., no treatment by subject interaction was postulated; Schmidt & Hunter, 2015; pp. 350–351), and we imposed the pre-post-test correlation to be *r*_*xx*_ = *r* = .600.

When the study implemented an only-post-test design (i.e., no pre-test assessment) we used the following formulas for effect size and sampling error variance, respectively:

4 $$ g=\frac{M_{e\_ post}-{M}_{c\_ post}}{S{D_{pooled}}_{pre}}\times \left(1-\frac{3}{\left(4\times N\right)-9}\right) $$

5 $$ Va{r}_g=\frac{N-1}{N-3}\times \frac{4}{N}\times \left(1+\frac{d^2}{8}\right)\times {\left(1-\frac{3}{\left(4\times N\right)-9}\right)}^2 $$

Finally, in a few cases, *t*- and *F*-values were used to calculate *d* (for the details, see the Supplemental Online Materials).

### Modeling approach

*Robust variance estimation* (RVE) with correlational weights was employed to perform the intercept and meta-regression models (Hedges, Tipton, & Johnson, 2010; Tanner-Smith, Tipton, & Polanin, 2016). RVE has been designed to model nested effect sizes (i.e., extracted from the same study). Two indexes were used to report the models’ between-cluster true (i.e., not due to random error) heterogeneity: *τ*^*2*^, which indicates the absolute amount of true heterogeneity; and *I*^*2*^, which indicates the percentage of true heterogeneity. In addition, we manipulated the within-study effect-size correlation (*ρ*) assumed by the RVE models to test the sensitivity of the results to this parameter. We performed these analyses with the Robumeta R package (Fisher, Tipton, & Zhipeng, 2017).

### Publication bias

We examined publication bias with two methods: Duval and Tweedie’s (2000) trim-and-fill analysis and Vevea and Woods’ (2005) selection models. The trim-and-fill method estimates whether some smaller-than-average effect sizes have been suppressed from the literature and calculates an adjusted overall effect size and standard error. This analysis was conducted after averaging the statistically dependent effects using Cheung and Chan’s (2014) approach. We employed the *L0* and *R0* estimators designed by Duval and Tweedie (2000). Vevea and Woods’ (2005) selection models estimate publication bias and calculate an adjusted overall effect size (but no standard error) by assigning to *p*-value ranges different weights. In other words, the method assumes that the probability of an effect not to be suppressed is a function of its *p*-value. As recommended by Pustejovsky and Rodgers (2019), the weights used in the publication bias analyses were not a function of the effect sizes (for more details, see Appendices C and D in the Supplemental Online Materials). We performed these analyses with the Metafor R package (Viechtbauer, 2010).

### True heterogeneity and sensitivity analysis

Explaining between-study true heterogeneity is one of the main goals of meta-analysis. While small to null true heterogeneity indicates that between-study differences are merely an artifact of random error (Schmidt, 2010), large amounts of true heterogeneity suggest that more than one true effect is present in the data. Moreover, true heterogeneity reduces the statistical power of meta-analytic models, tends to artificially inflate overall effect sizes in asymmetric distributions, and sometimes produces biased publication-bias adjusted estimates (Cheung & Chan, 2014; Henmi & Copas, 2010; Schmidt & Hunter, 2015; Stanley, 2017).

Investigating the sources of true heterogeneity is thus essential to make the results more interpretable and accurate. Therefore, beyond running meta-regression analysis, we performed a two-step sensitivity analysis. First, we excluded three studies that, probably due to lack of random allocation or small sample sizes, reported unusually high between-group differences (≈ 1 SD) in the participants’ baseline IQ (Patscheke, Degé, & Schwarzer, 2019; Roden, Kreutz, & Bongard, 2012; Roden, Grube, Bongard, & Kreutz, 2014). That is, these three studies were included in the main analysis but removed from the sensitivity analysis. Such large baseline differences make any findings hard to interpret and may introduce noise in the data. Second, we ran Viechtbauer and Cheung’s (2010) influential case analysis. This method evaluates whether some effect sizes exerted an unusually strong influence on the model’s parameters such as the amount of between-study true heterogeneity (*τ*^*2*^). Those effect sizes that inflated true heterogeneity were excluded.

### Bayesian analysis

A vast quantity of data regarding cognitive-training programs has been collected in the last 15 years. For example, Sala et al.’s (2019a) second-order meta-analysis estimates that more than 20,000 participants have undergone cognitive-training programs such as music training, videogame training, and WM training. This previous evidence can be employed to establish a set of distributional assumptions (informative priors) in the Bayesian framework.

The distribution of the effect sizes was assumed to be normal. Based on Sala et al.’s (2019a) second-order meta-analysis, we expected the mean effect size to be null (prior $\overline{g}$ = 0) in models including active control groups and slightly positive (prior $\overline{g}$ = 0.150) in models including passive controls groups. The prior for the standard deviation was the same in all the models (*SD*_*g*_ = 0.500). The true heterogeneity parameter (*τ*) was assumed to have a half-Cauchy distribution (centered on 0 and scale γ = 10) in all the models. No further prior was used for other moderators.

We thus estimated the Bayes factors (*BF*s) for two sets of competing hypotheses for $\overline{g}$ and *τ*. First, we compared the alternative hypothesis H1: $\overline{g}$ ≠ 0 with the null hypothesis H0: $\overline{g}$ = 0. Second, we compared the alternative hypothesis H1: *τ* > 0 with the null hypothesis H0: *τ* = 0. *BF*s > 1 indicated support for H1, while *BF*s < 1 indicated support for H0. In line with common guidelines, H1 was considered as substantially supported only if BF > 3 (i.e., H1 three times more likely to be true than H0; e.g., Dougherty, Hamovitz, & Tidwell, 2016). Analogously, H0 was substantially supported only if *BF* < 0.333 (i.e., H0 three times more likely to be true than H1). Since the priors were conditional to the type of controls employed by the primary study (as indicated by Sala et al., 2019a), these analyses were carried out after running moderator analysis. The analyses were carried out with the bayesmeta R package (Röver, 2017).

## Results

### Descriptive statistics

The mean age of the samples was 6.45 years. The median age was 5.90, the first and third quartiles were 5.03 and 7.85, and the mean age range was 3.50–11.59. The mean Baseline difference was -0.038, the median was 0, the first and third quartiles were -0.210 and 0.141, and the range was -1.058–0.844. The mean duration of training was 53.37 h (range 2.00–507.00, median 30.00), 29.29 weeks (range 3.00–117.00, median 26.00), and 53.43 sessions (range 6.00–195.00, median 30.00). The descriptive statistics of the categorical moderators are reported in Table 1.

**Table 1:** Number of studies and effect sizes sorted by categorical moderators

Moderator	No. of studies	No. of effect sizes
Randomization		
Non-random	33	139
Random	23	115
Control group		
Non-active	41	144
Active	23	110
Outcome measures		
Memory	19	57
Verbal	33	89
Non-verbal	27	69
Speed	13	39

### Main analyses

The overall effect size of the RVE intercept model was $\overline{g}$ = 0.184, *SE* = 0.041, 95% confidence interval (CI) [0.101; 0.268], *m* = 54, *k* = 254, *df* = 38.36, *p* < .001, *τ*^*2*^ = 0.041, *I*^*2*^ = 43.16%. Different values of within-study effect-size correlation (*ρ*) did not significantly affect the results ($\overline{g}$ range 0.184– 0.185, *τ*^*2*^ = 0.041). The random-effect (RE) model (with Cheung and Chan’s correction) yielded very similar estimates: $\overline{g}$ = 0.176, *SE* = 0.037, *p* < .001, *τ*^*2*^ = 0.033. Overall, the results showed a small and moderately heterogeneous overall effect of music training on cognitive and academic outcomes. The results were not affected by modeling choices (i.e., *ρ* values and procedure for modeling nested data).

Baseline difference and Type of controls were the only two statistically significant moderators (*p* = .031 and *p* = .035, respectively) and accounted for part of the true heterogeneity (*τ*^*2*^ = 0.038, *I*^*2*^ = 34.87%). Age was not significant (*p* = .403), neither was Allocation (*p* = .518). No significant differences were found across the four broad groups of outcome measures (all *p*s ≥ .624; Holm’s correction for multiple comparisons), nor across the more fine-grained categorization (eight levels, all *p*s ≥ .362), and there was no difference between cognitive skills and measures of academic achievement (*p* = .981). Duration of training was not significant either (*p* = .266, *p* = .952, and *p* = .662 for hours, weeks, and sessions, respectively).

#### Type of controls

Since Type of controls was statistically significant, we performed the analyses on the two sub-samples separately. In those studies that implemented non-active controls, the results showed a small and moderately heterogeneous overall effect of music training on cognitive and academic outcomes. The overall effect size was $\overline{\boldsymbol{g}}$ = 0.228, SE = 0.045, 95% CI [0.137; 0.320], m = 41, k = 144, df = 30.1, p < .001, τ^2^ = 0.042, I^2^ = 43.11%. Different values of within-study effect-size correlation (ρ) did not affect the results ($\overline{\boldsymbol{g}}$ = 0.228, τ^2^ = 0.042). The RE model provided similar results, $\overline{\boldsymbol{g}}$ = 0.201, SE = 0.041, p < .001, τ^2^ = 0.023. Again, the results were not affected by modeling choices. Also, some evidence of a small publication bias was found. The trim-and-fill retrieved no missing study with the L0 estimator. Five missing studies were retrieved with the R0 estimator, and the adjusted estimate was $\overline{\boldsymbol{g}}$ = 0.170, 95% CI [0.064; 0.276]. Vevea and Woods’ (2005) selection model calculated a similar estimate ($\overline{\boldsymbol{g}}$ = 0.119). Finally, the Bayes factors confirmed these findings. BF_g_ was greater than 730,000, indicating that $\overline{\boldsymbol{g}}$ was far more likely to be non-null (H1: $\overline{\boldsymbol{g}}$ ≠ 0) than null (H0: $\overline{\boldsymbol{g}}$ = 0). Regarding the model’s true heterogeneity, BF_τ_ was greater than 5,000, again indicating that τ was far more likely to be positive than null.

In those studies that implemented active controls, the results showed a near-zero and slightly heterogeneous overall effect of music training on cognitive and academic outcomes. The overall effect size was $\overline{g}$ = 0.056, *SE* = 0.058, 95% CI [-0.069; 0.182], *m* = 23, *k* = 110, *df* = 12.6, *p* = .350, *τ*^*2*^ = 0.025, *I*^*2*^ = 23.10%. Different values of within-study effect-size correlation (*ρ*) did not significantly affect the results ($\overline{g}$ range 0.054–0.057, *τ*^*2*^ range 0.023–0.025). The results were robust to the type of modeling approach employed. In fact, the RE model provided similar results, $\overline{g}$ = 0.090, *SE* = 0.060, *p* = .136, *τ*^*2*^ = 0.032. Some evidence of a small publication bias was found, suggesting that the unbiased overall effect size is essentially null. No missing study was retrieved with the *L0* estimator, whereas the *R0* estimator identified four missing studies and the adjusted estimate was $\overline{g}$ = -0.020, 95% CI [-0.183; 0.142]. The selection model estimate was $\overline{g}$ = 0.039. The Bayes factors were *BF*_*g*_ = 1.231 and *BF*_*τ*_ = 0.044. These results showed that – as indicated by the publication-bias-corrected estimates – $\overline{g}$ was not convincingly more likely to be non-null than null (*BF*_*g*_ < 3), and that *τ* was approximately 23 times more likely to be null than positive. The latter finding confirms that the low observed true heterogeneity (*τ*^*2*^ = 0.025, *I*^*2*^ = 23.10%) is very likely to be spurious.

### Sensitivity analyses

This section replicated the analyses after excluding the three studies reporting large baseline IQ differences across the groups and implemented Viechtbauer and Cheung’s (2010) influential case analysis to explain the model’s residual true heterogeneity (if any). The overall effect size of the RVE intercept model was $\overline{g}$ = 0.166, *SE* = 0.041, 95% CI [0.083; 0.249], *m* = 51, *k* = 235, *df* = 34.9, *p* < .001, *τ*^*2*^ = 0.036, *I*^*2*^ = 40.62%. Different values of within-study effect-size correlation (*ρ*) did not significantly affect the results ($\overline{g}$ range 0.165–0.166, *τ*^*2*^ range 0.035–0.036). The random-effect (RE) model provided similar estimates: $\overline{g}$ = 0.149, *SE* = 0.035, *p* < .001, *τ*^*2*^ = 0.024. Baseline difference and Type of controls were again the only two statistically significant moderators (*p* = .017 and *p* = .003, respectively) and accounted for part of the true heterogeneity (*τ*^*2*^ = 0.029, *I*^*2*^ = 29.70%). Therefore, the results were pretty much the same as in the main analyses so far.

#### Non-active controls

When non-active controls were used, the overall effect size was $\overline{g}$ = 0.226, *SE* = 0.045, 95% CI [0.133; 0.319], *m* = 40, *k* = 139, *df* = 29.2, *p* < .001, *τ*^*2*^ = 0.041, *I*^*2*^ = 42.96%. Different values of within-study effect-size correlation (*ρ*) did not significantly affect the results ($\overline{g}$ = 0.226, *τ*^*2*^ = 0.041). The RE model provided similar results, $\overline{g}$ = 0.200, *SE* = 0.041, *p* < .001, *τ*^*2*^ = 0.024. Five effect sizes were found to be significantly inflating the true heterogeneity. After excluding these effect sizes, the overall effect size was $\overline{g}$ = 0.181, *SE* = 0.042, 95% CI [0.093; 0.268], *m* = 39, *k* = 134, *df* = 21.9, *p* < .001, *τ*^*2*^ = 0.018, *I*^*2*^ = 24.92%. Similar results were obtained with the RE model, $\overline{g}$ = 0.161, *SE* = 0.037, *p* < .001, *τ*^*2*^ = 0.013.

Finally, in order to investigate the sources of the unexplained true heterogeneity (*τ*^*2*^ = 0.018, *I*^*2*^ = 24.92%), a moderator analysis was run. Randomization was the only statistically significant moderator (*p* = .042) and explained nearly all the true heterogeneity (*τ*^*2*^ = 0.005, *I*^*2*^ = 7.61%). Therefore, the observed true between-study heterogeneity in the studies employing non-active controls was accounted for by a few extreme effect sizes and the type of allocation of participants to the groups. For non-randomized studies, the overall effect sizes were $\overline{g}$ = 0.246, *SE* = 0.049, 95% CI [0.140; 0.352], *p* < .001; for randomized studies, the relevant statistics were $\overline{g}$ = 0.064, *SE* = 0.065, 95% CI [-0.116; 0.244], *p* = .381. Thus, when random allocation was employed, the overall effect was near-zero.

##### Publication bias analysis: Studies without randomization

With the studies that did not implement any randomization of participants’ allocation, the trim-and-fill analysis retrieved two missing studies with the *L0* estimator (adjusted estimates $\overline{g}$ = 0.211, 95% CI [0.095; 0.328]). Three missing studies were retrieved with the *R0* estimator (adjusted estimates $\overline{g}$ = 0.189, 95% CI [0.068; 0.310]). Vevea and Woods’ (2005) selection model calculated a more conservative estimate ($\overline{g}$ = 0.126). Thus, a small amount of publication bias was still detected. The Bayes factors were *BF*_*g*_ = 217.840 ($\overline{g}$ far more likely to be non-null than null) and *BF*_*τ*_ = 0.021 (*τ* nearly 50 times more likely to be null than positive). While confirming that the overall effect size of music training in non-randomized samples and passive controls is positive (yet small), these results showed that no between-study true heterogeneity was present in the data.

##### Publication bias analysis: Studies with randomization

Regarding the randomized samples, all the publication bias analyses estimated a substantially null overall effect. The trim-and-fill analysis retrieved six and ten studies with the *L0* and *R0* estimators, respectively (adjusted estimates $\overline{g}$ = 0.009, 95% CI [-0.095; 0.113] and $\overline{g}$ = -0.034, 95% CI [-0.131; 0.063]). Vevea and Woods’ (2005) selection model yielded a similar estimate ($\overline{g}$ = -0.002). The Bayes factors were *BF*_*g*_ = 0.257 and *BF*_*τ*_ = 0.025. Therefore, the Bayes factors provided compelling evidence that both $\overline{g}$ and *τ* are more likely to be null than non-null (approximatively 4 and 40 times, respectively).

#### Active controls

Turning our attention to the studies implementing active controls, the overall effect size was $\overline{g}$ = -0.021, *SE* = 0.032, 95% CI [-0.109; 0.068], *m* = 20, *k* = 96, *df* = 4.2, *p* = .558, *τ*^*2*^ = 0, *I*^*2*^ = 0%. Different values of within-study effect-size correlation (*ρ*) did not affect the results ($\overline{g}$ = -0.021, *τ*^*2*^ = 0). The RE model provided similar results, $\overline{g}$ = -0.010, *SE* = 0.035, *p* = .787, *τ*^*2*^ = 0. Since this model showed no true heterogeneity and null overall effects, no publication bias analysis was performed. The Bayes factors largely favored the null hypothesis (*BF*_*g*_ = 0.063 and *BF*_*τ*_ = 0.006). The null hypothesis was approximatively 16 times and 180 times more likely than the alternative hypothesis for $\overline{g}$ and *τ*, respectively. In brief, all the analyses showed that the overall effect in studies implementing active controls is null and homogeneous across studies (i.e., $\overline{g}$ = 0, *τ*^*2*^ = 0).

## Discussion

This meta-analytic review investigated the impact of music training on children’s cognitive skills and academic achievement. The overall impact of music training programs on cognitive and academic outcomes is weak and moderately heterogeneous ($\overline{g}$ = 0.184, *SE* = 0.041, *τ*^*2*^ = 0.041, *I*^*2*^ = 43.16%). The inspection of true heterogeneity shows that there is an inverse relationship between the studies’ design quality and magnitude of the effect sizes. Specifically, those studies using active controls or implementing random assignment report homogeneous null or near-zero effects ($\overline{g}$ = -0.021–0.064, *τ*^*2*^ ≤ 0.005). Conversely, a small overall effect size is observed in those studies employing neither active controls nor random assignment ($\overline{g}$ = 0.246). The results of the Bayesian analyses corroborate the conclusions that the unbiased effect of music training on cognitive and academic skills is null and highly consistent across the studies (i.e., $\overline{g}$ = 0 and *τ*^*2*^ = 0). No other study features (e.g., age, duration of training, and outcome measure) seem to have any influence on the effect sizes – not even the outcome measures. In particular, contrary to Cooper’s (2019) hypothesis, there was no difference between cognitive skills and academic achievement (literacy and mathematics), which means that it is justifiable to pool the two outcomes together, as was done for example in Sala and Gobet (2017b). Altogether, these results indicate that music training fails to produce solid improvements in all the examined cognitive and academic skills equally. Finally, only a low amount of publication bias is observed in the models (about 0.100 standardized mean difference at most), which is in line with the near-zero effect sizes estimated. The results are summarized in Table 2.

**Table 2:** Overall effects in the meta-analytic models

Model (1)	$\overline{g}$.RVE (SE) (2)	Adj.$\overline{g}$ (range) (3)	Heterogeneity (4)	Residual heterogeneity (5)	Bayes factors (6)
Main analyses					
Overall	0.184 (0.041)	–	*τ*^*2*^ = 0.041, *I*^*2*^ = 43.16	*τ*^*2*^ = 0.038, *I*^*2*^ = 34.87	–
Non-active	0.228 (0.045)	0.119 – 0.228	*τ*^*2*^ = 0.042, *I*^*2*^ = 43.11	–	*BF*_*g*_ > 7.3×10^5^, *BF*_*τ*_ > 5×10^3^
Active	0.056 (0.058)	-0.020 – 0.056	*τ*^*2*^ = 0.025, *I*^*2*^ = 23.10	–	*BF*_*g*_ = 1.231, *BF*_*τ*_ = 0.044
Sensitivity analyses					
Overall	0.166 (0.041)	–	*τ*^*2*^ = 0.036, *I*^*2*^ = 40.62	*τ*^*2*^ = 0.029, *I*^*2*^ = 29.70	–
Non-active	0.226 (0.045)	–	*τ*^*2*^ = 0.041, *I*^*2*^ = 42.96	*τ*^*2*^ = 0.005, *I*^*2*^ = 7.606	–
Non-random	0.246 (0.049)	0.126 – 0.211	–	–	*BF*_*g*_ = 217.840, *BF*_*τ*_ = 0.021
Random	0.064 (0.065)	-0.034 – 0.009	–	–	*BF*_*g*_ = 0.257, *BF*_*τ*_ = 0.025
Active	-0.021 (0.032)	–	*τ*^*2*^ = 0.000, *I*^*2*^ = 0.000	–	*BF*_*g*_ = 0.063, *BF*_*τ*_ = 0.006

*Note*. (1) The meta-analytic model; (2) the overall RVE effect size (Standard Error); (3) the range of the publication bias adjusted estimates; (4) the amount of true heterogeneity of the model; (5) the true heterogeneity after running meta-regression (and sensitivity analysis); (6) Bayes factors comparing the alternative hypotheses (H1: $\overline{g}$ ≠ 0; H1: *τ* > 0) with the null hypotheses (H0: $\overline{g}$ = 0; H0: *τ* = 0)

These findings confirm and extend the conclusions of the previous comprehensive meta-analysis in the field (Sala & Gobet, 2017b). Along with re-establishing the fundamental role of design quality in affecting the experimental results, the present meta-analysis has succeeded in explaining *all* the observed true heterogeneity. We can thus conclude that these findings convincingly refute all the theories claiming that music training *causes* improvements in any domain-general cognitive skill or academic achievement (e.g., Moreno et al., 2011; Patel, 2011; Saarikivi et al., 2019; Tierney & Kraus, 2013). In fact, there is no need to postulate any explanatory mechanism in the absence of any genuine effect or between-study variability. In other words, since there is no phenomenon, there is nothing to explain. More broadly, these results establish once again that far transfer – due to the very nature of human cognition – is an extremely rare occurrence (Gobet & Simon, 1996; Sala & Gobet, 2019).

### Beyond meta-analytic evidence

It is worth noting that other researchers have reached the same conclusions using different methodologies. To begin with, Mosing, Madison, Pedersen, and Ullén (2016) have investigated the relationship between music training and general intelligence in twins. Notably, music-trained twins do not possess a higher IQ than non-music-trained co-twins. This study thus suggests that engaging in music has no effect on people’s IQ. Swaminathan, Schellenberg, and Khalil (2017) show that music aptitude, rather than the amount of music training, predicts fluid intelligence in a sample of adults. This finding upholds the idea that the correlation between intelligence and engagement in music is mediated by innate (as opposed to trained) music skills. Similarly, Swaminathan, Schellenberg, and Venkatesan (2018) demonstrate that the correlation between amount of music training and reading ability in adults disappears when domain-general cognitive skills are controlled for.

These findings corroborate the hypothesis according to which the observed correlation between music training and particular domain-general cognitive/academic skills is a byproduct of previous abilities. Once pre-existing differences in overall cognitive function are ruled out, the correlation disappears (Swaminathan & Schellenberg, 2019). Therefore, there is no reason to support the hypothesis that music training boosts cognition or academic skills. Rather, all the evidence points toward the opposite conclusion, that is, that the impact of music training on cognitive and academic skills is null.

Finally, the failure of music-training regimens to induce any generalized effect is mirrored by findings in other cognitive-training literatures. For instance, WM training does not enhance children’s domain-general cognitive skills or academic achievement (Aksayli, Sala, & Gobet, 2019; Melby-Lervåg et al., 2016; Sala & Gobet, 2020). The same applies to action and nonaction videogame training and brain training (Duyck & Op de Beeck, 2019; Kassai, Futo, Demetrovics, & Takacs, 2019; Libertus et al., 2017; Lintern & Boot, 2019; Sala et al., 2019a; Sala, Tatlidil, & Gobet, 2018, 2019b; Simons et al., 2016).

### The perception of music training effectiveness is biased

It is our conviction that, while the data show a consistent picture, the narrative that has been built around music training is substantially distorted. For example, Schellenberg (2019) has shown how correlational evidence is often used by scholars to incorrectly infer causal relationships between engagement in music and non-music outcomes. Correlation is notoriously insufficient to establish causal links between variables, which makes Schellenberg’s (2019) findings quite concerning. Interestingly, this problem appears to be particularly severe in neuroscientific studies.

The overall interpretation of the results reported in the primary studies is another example of the extent to which authors sometimes misread the empirical evidence presumably supporting music training. For instance, Barbaroux et al.’s (2019) study does not implement any type of controls, which makes their results uninterpretable. Tierney et al. (2015) report non-significant and inconsistent effects on language-related outcomes between a music training group and an active control group. However, this study is not experimental because the participants were recruited after they had chosen what activity to take part in (i.e., self-selection of the sample). (This is why, incidentally, this study is not included in the present meta-analysis.) Despite representing very little evidence in favor of a causal link between music training and improved cognitive/academic skills, the study has gained a considerable amount of attention in news outlets and among researchers in the field (top 5% in Altmetric). In the same vein, Nan et al. (2018) have found no significant effect of music training on any two music-related measures and no effect at all on the examined non-music outcome measures. (The paper reports a barely significant effect [*p* = .044] in an auditory task that is obtained with an ANOVA performed on the mean pre-post-test gains. This is a well-known incorrect practice that inflates Type I error rates.) Therefore, this study corroborates the idea that the impact of music training on cognitive/academic skills is slim to null. Nonetheless, both the authors and several news outlets provide an over-optimistic, if not utterly incorrect, view of the benefits of music training (e.g., McCarthy, 2018).

By contrast, the two largest randomized controlled trials in the field have been either somewhat ignored (Aleman et al., 2017) or nearly completely overlooked (Haywood et al., 2015) by researchers involved in music training (and news outlets). Both studies report no effect of music training on any cognitive or academic skills. Neither of them makes any overstatement about the benefits of music training on any domain-general cognitive or academic skill. It is thus apparent that if *all* the results are considered and correctly interpreted, the whole music training literature depicts a very consistent mosaic. What is mixed is how the same findings are described by different scholars (Schmidt, 2017).

### Conclusions and recommendations for future research

This meta-analysis has examined the experimental evidence regarding the impact of music training on children’s non-music cognitive skills and academic achievement. The ineffectiveness of the practice is apparent and highly consistent across studies. Moreover, recent correlational studies have confirmed that music engagement is not associated with domain-general cognitive skills or academic performance.

Two alternative potential avenues involving music activities may be worth some interest. First, music may be beneficial for non-cognitive constructs in children such as prosocial behavior and self-esteem (e.g., Aleman et al., 2017). These possible advantages are not likely to be specific to music, though. In fact, any enticing and empowering activity may improve children’s well-being. Second, elements of music instruction (e.g., arithmetical music notation) could be used to facilitate learning in other disciplines such as arithmetic (Azaryahu, Courey, Elkoshi, & Adi-Japha, 2019; Courey, Balogh, Siker, & Paik, 2012; Ribeiro & Santos, 2017). Too few studies have been conducted to reach a definite conclusion. Nonetheless, this approach is undoubtedly more likely to succeed than the music training programs reviewed in this meta-analysis. In fact, while the latter program regimens have tried and failed to reach cognitive enhancement via music training, the former methodology tries to convey domain-specific knowledge by focusing on domain-specific information. This type of near transfer is notoriously much easier to achieve (Gobet, 2016; Gobet & Simon, 1996).

## Electronic supplementary material

ESM 1 (DOCX 39 kb)

## Footnotes

1 Here, the adjective “multilevel” broadly refers to any technique that allows the researcher to correctly model multivariate data (i.e., effect sizes) nested within studies. We do not intend to imply that the specific modeling method used in the present meta-analysis is superior to other methods. Rather, we simply highlight the necessity of adopting multilevel/multivariate techniques in order to produce accurate results.
